# Testing, explaining, and exploring models of facial expressions of emotions

**DOI:** 10.1126/sciadv.abq8421

**Published:** 2023-02-10

**Authors:** Lukas Snoek, Rachael E. Jack, Philippe G. Schyns, Oliver G. B. Garrod, Maximilian Mittenbühler, Chaona Chen, Suzanne Oosterwijk, H. Steven Scholte

**Affiliations:** ^1^ Department of Psychology, University of Amsterdam, Amsterdam, Netherlands.; ^2^ School of Psychology and Neuroscience, University of Glasgow, Glasgow, UK.; ^3^ Department of Computer Science, University of Tübingen, Tübingen, Germany.

## Abstract

Models are the hallmark of mature scientific inquiry. In psychology, this maturity has been reached in a pervasive question—what models best represent facial expressions of emotion? Several hypotheses propose different combinations of facial movements [action units (AUs)] as best representing the six basic emotions and four conversational signals across cultures. We developed a new framework to formalize such hypotheses as predictive models, compare their ability to predict human emotion categorizations in Western and East Asian cultures, explain the causal role of individual AUs, and explore updated, culture-accented models that improve performance by reducing a prevalent Western bias. Our predictive models also provide a noise ceiling to inform the explanatory power and limitations of different factors (e.g., AUs and individual differences). Thus, our framework provides a new approach to test models of social signals, explain their predictive power, and explore their optimization, with direct implications for theory development.

## Teaser

A framework evaluates facial expression models, reveals their Western bias, and develops better, culture-accented models.

## INTRODUCTION

In mature scientific endeavors, models are used to advance knowledge in three complementary ways: by predicting a phenomenon, explaining its causes, and, using the enhanced understanding derived from these explanations, exploring improved models of the phenomenon (*1*, *2*). The field of psychology provides a strong case that exemplifies the development of models to explain a central human behavior: the recognition of emotions from facial expressions. Since Darwin’s seminal work on the evolutionary origins of facial expressions (*3*), several other models of facial expressions have been proposed as more accurate representations of the six classic basic emotions: anger, disgust, fear, happy, sadness, and surprise [reviewed in (*4*)]. Researchers often use an influential taxonomy of human facial movements—the Facial Action Coding System (FACS) (*5*)—to operationalize facial expressions as combinations of unitary facial movements called “action units” (AUs). Then, models of the basic emotions become hypotheses about which AU combinations represent each category. For example, Ekman and Friesen (*5*) describe the facial expression of anger as comprising Brow Lowerer (AU4), Upper Lid Raiser (AU5), Lid Tightener (AU7), and Lip Tightener (AU23), whereas Cordaro and colleagues (*6*) describe the same facial expression as comprising only Brow Lowerer (AU4) and Lid Tightener (AU7). Here, a model thus takes as inputs the AUs that make up the facial expression—e.g., AU4, AU5, AU7, and AU23—and, from these, predicts the associated emotion category as output (e.g., “anger”).

**Fig. 1.**
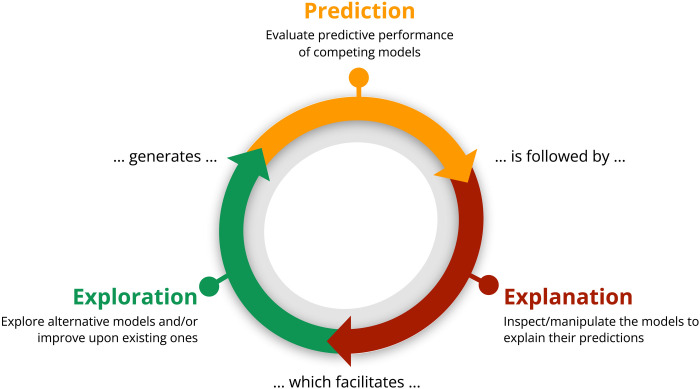
The prediction-explanation-exploration framework. At prediction, models generate predictions to compare to actual behavior. Next, at explanation, models are experimentally manipulated to understand the causal influences of their components (e.g., here, individual facial movements called AUs). Consequently, at exploration, the explanatory insights derived from the explanation stage guide the automatic construction of alternative and improved models, thus completing the cycle.

The search for representative models of facial expressions of emotion has been a long-lasting endeavor that has generated many competing models (*3*, *5*–*7*). However, these models often remain qualitative, descriptive hypotheses of how AUs relate to emotions, making them difficult to quantitatively evaluate and compare. Using a novel technique, “hypothesis kernel analysis,” we aim to improve such qualitative hypotheses by turning them into formal models that can generate quantitative predictions of the emotions associated with a given facial expression. We further propose a new prediction-explanation-exploration framework (see Fig. 1). This framework provides a principled and general approach to evaluate, compare, and improve the predictive performance and limitations of predictive models, including models of facial expressions of emotion.

Our framework quantifies how well different models predict human emotion categorizations, explains their predictions by identifying the specific AUs that are critical (or detrimental) to categorization performance, and uses this information to explore updated AU-based models that improve performance. Here, we used this framework to systematically compare and quantitatively evaluate six influential AU-based models of facial expressions of the six classic basic emotions [reviewed in (*4*)] and a data-driven model. We extend the comparisons and evaluations across Western (WE) and East Asian (EA) cultures to construct improved, culturally aware models. To highlight the robustness of our approach, we furthermore generalize our framework beyond the basic six emotions to models of four conversational signals.

### The prediction-explanation-exploration framework

Figure 1 illustrates this new framework, which outlines how to evaluate, explain, and optimize models throughout three stages: prediction, explanation, and exploration. First, the prediction stage generates model predictions (here, categorizations of emotions) and compares these with human categorizations of the same data, resulting in a model performance score that summarizes how accurately model predictions align with human categorization behavior. In the second explanation stage, the constituting elements of the model (here, individual facial movements—AUs) are systematically manipulated to evaluate their causal effects on behavioral prediction and how they affect model categorization performance. In the last exploration stage, the causal effects estimated from the explanation stage are used to automatically construct updated and improved models that comprise new hypotheses (here, about the specific AUs that represent each of the six emotions, including culture-specific accents). These new, optimized models benefit from the insights gained from the entire set of model comparisons, thus effectively combining their relative strengths to develop knowledge under this new epistemology.

**Fig. 2.**
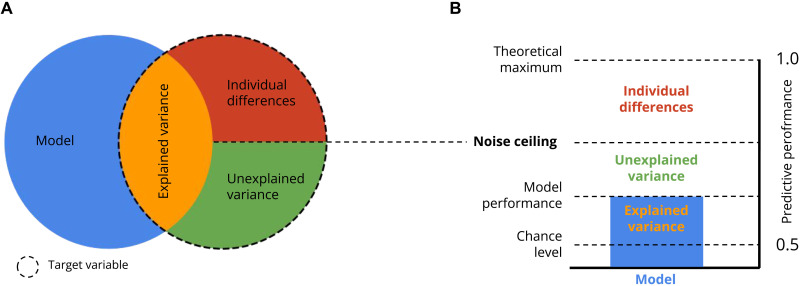
Variance partitioning of emotion categorizations. (**A**) The predictions of a given model (blue circle) that match the target variable (dashed circle) represent the “explained variance” (represented in orange). The noise ceiling (horizontal dashed line) further divides the leftover variance (red + green set) into individual differences (red) and unexplained variance (green). Thus, individual differences cannot, in principle, be explained by any fixed model. (**B**) Variance partitioning of the target variable is represented as a bar graph. The noise ceiling partitions the difference between the explained variance (orange) and the theoretical maximum into the unexplained variance (represented in green; noise ceiling minus model performance) and individual differences (represented in red; theoretical maximum performance minus noise ceiling).

A notable advantage of predictive models is that they can decompose variance in human categorization behavior into three distinct components (see Fig. 2), providing insight into the model’s limitations. The first component is the explained variance (represented in orange)—here, the proportion of variance in human categorization behavior that is correctly predicted by a facial expression model. The other two components are determined by the model’s noise ceiling (*8*, *9*), which subdivides the remaining variance into unexplained variance (represented in green) and individual differences (represented in red), which arise from individuals who categorize the same facial expressions differently. Thus, the noise ceiling emphasizes the notion that a single “universal” model cannot, by definition, explain the variations in categorization behavior between individuals and therefore represents the maximum performance of any model that ignores these individual differences. Here, we use noise ceilings to provide an upper limit of performance of such fixed AU-based models of facial expressions.

To apply the prediction-explanation-exploration framework to our selection of models of the six classic emotions, we turned their descriptive hypotheses into predictive models using a new methodology (see the “Hypothesis kernel analysis” section). We then quantitatively evaluated, compared, and optimized these predictive models within the prediction-explanation-exploration framework.

### Modeling human categorization of facial expressions of emotions within the prediction-explanation-exploration framework

Using the prediction-explanation-exploration framework, we tested seven influential models of facial expressions of the six classic emotions (*3*, *5*–*7*, *10*, *11*). We selected these models based on their explicit hypotheses about the specific AUs associated with each of the six classic emotions [summarized in (*4*); see Table 1]. We preview our results according to the three main stages of our framework.

**Table 1.** Evaluated basic emotion models in our study. We took the mappings of Darwin (*3*) from Matsumoto (*10*). Both the “reference configuration” (ref.) and the “international core pattern” (ICP) from Cordaro *et al.* (*6*) are included. The + symbol means that AUs occur together. AUs following a comma represent optional AUs. The ∨ symbol represents “or,” so, e.g., (25 ∨ 26) means that either AU25 or AU26 may be included in the configuration. When multiple configurations are explicitly proposed for a given emotion (i.e., a “many-to-one” mapping), they are represented as separate bullet points. For the Jack *et al.* (*11*) model, only AUs included in >50% of the participants are listed.

Emotion category	Darwin [1872; (*3*)]	Friesen and Ekman [1978;(*5*)]	Matsumoto *et al.* [2008; (*10*)]	Cordaro *et al.* [2018; ref. (*6*)]	Cordaro *et al.* [2018; ICP (*6*)]	Keltner *et al.* [2019; (*7*)]	Jack *et al.* [2014; (*11*)]
Anger	4 + 5 + 24 + 38	• 4 + 5 + 7 + 10 + 22 + 23 + (25 ∨ 26)	4 + (5 ∨ 7) + 22 + 23 + 24	4 + 5 + 7 + 23	4 + 7	4 + 5 + 17 + 23 + 24	9 + 10 + 16 + 22
		• 4 + 5 + 7 + 10 + 23 + (25 ∨ 26)					
		• 4 + 5 + 7 + 17 + (23 ∨ 24)					
		• 4 + 5 + 7 + (23 ∨ 24)					
		• 4 + (5 ∨ 7)					
		• 17 + 24					
Disgust	10 + 16 + 22 + 25 + 26	• (9 ∨ 10) + 17	(9 ∨ 10), (25 ∨ 26)	9 + 15 + 16	4 + 6 + 7 + 9 + 10 + 25 + (26 ∨ 27)	7 + 9 + 19 + 25 + 26	9 + 10 + 11 + 43
		• (9 ∨ 10)					
		• (9 ∨ 10) + 16 + (25 ∨ 26)					
Fear	1 + 2 + 5 + 20	• 1 + 2 + 4	1 + 2 + 4 + 5 + 20, (25 ∨ 26)	1 + 2 + 4 + 5 + 20 + 25 + 26	1 + 2 + 5 + 7 + 25 + (26 ∨ 27)	1 + 2 + 4 + 5 + 7 + 20 + 25	4 + 5 + 20
		• 1 + 2 + 4 + 5 + 20 + (25 ∨ 26 ∨ 27)					
		• 1 + 2 + 4 + 5 + (25 ∨ 26 ∨ 27)					
		• 1 + 2 + 4 + 5					
		• 1 + 2 + 5 + (25 ∨ 26 ∨ 27)					
		• 5 + 20 + (25 ∨ 26 ∨ 27)					
		• 5 + 20					
		• 20					
Happy	6 + 12	• 12	6 + 12	6 + 12	6 + 7 + 12 + 16 + 25 + (26 ∨ 27)	6 + 7 + 12 + 25 + 26	6 + 12 + 13 + 14 + 25
		• 6 + 12					
Sadness	1 + 15	• 1 + 4	1 + 15, 4, 17	1 + 4 + 5	4 + 43	1 + 4 + 6 + 15 + 17	4 + 15 + 17 + 24 + 43
		• 1 + 4 + (11 ∨ 15)					
		• 1 + 4 + 15 + 17					
		• 6 + 15					
		• 11 + 17					
		• 1					
Surprise	1 + 2 + 5 + 25 + 26	• 1 + 2 + 5 + (26 ∨ 27)	1 + 2 + 5 + (25 ∨ 26)	1 + 2 + 5 + 26	1 + 2 + 5 + 25 + (26 ∨ 27)	1 + 2 + 5 + 25 + 26	1 + 2 + 5 + 26 + 27
		• 1 + 2 + 5					
		• 1 + 2 + (26 ∨ 27)					
		• 5 + (26 ∨ 27)					

#### Prediction

We evaluated how each of the seven models predicted each basic emotion category using a large dataset of 2400 emotion categorization trials per participant. Each trial comprised an agnostically generated facial animation composed of a random combination of dynamic AUs. We instructed 60 WE participants and 60 EA participants to categorize each facial animation video as one of the six classic emotions—“happy,” “surprise,” “fear,” “disgust,” “anger,” or “sadness”—only if they perceived that the facial animation represented one of the emotions—or to select “do not know” if they did not (see Materials and Methods for details). We used the same trials to predict the most likely emotion category of each model to assess how well it predicts human emotion categorization behavior. We found that all seven models explain a substantial proportion of variations of human behavior, albeit below the noise ceiling, suggesting that each model can be further optimized to better fit human behavior. Furthermore, models performed better for WE than EA participants, suggesting that they are biased toward WE representations of facial expressions of emotions and lack important accents of EA culture.

#### Explanation

Next, to explain how each individual AU in each model contributed to emotion categorization performance, we used an “AU ablation” procedure that systematically removed individual AUs from each model and recomputed its prediction of human behavior, separately for WE and EA cultures. This procedure identified a set of culture-specific performance-critical AUs, which, when ablated, decrease prediction performance of the model. In other words, performance-critical AUs are necessary to accurately categorize each emotion among the five others. The procedure additionally identified a set of culture-specific performance-detrimental AUs, which, when ablated, increase prediction performance of the model. In other words, performance-detrimental AUs hinder accurate categorization of each emotion among the five others.

#### Exploration

Last, to explore whether the causal AUs that explain performance do improve predictions, we added performance-critical AUs to the original models and removed performance-detrimental AUs, separately for WE and EA cultures, thereby generating updated, optimized, and culture-specific models. We found that their prediction performance on new stimuli and participants (i.e., data not used in the prediction and explanation stages) improved substantially relative to the original models, removing the WE bias reported earlier. However, AU-enhanced models still performed below the noise ceiling, suggesting that models could improve by refining their AU representations (e.g., by considering the time course of AU activations) or by adding additional expressor-related features (e.g., the ethnicity of the face). Moreover, the substantial portion of variance due to individual differences suggests that models can benefit from additional perceiver-related characteristics beyond culture, such as sex or age.

### Generalization to other emotions

As the six basic emotions are only a subset of mental states that a face can express (*12*, *13*), we extended our framework to a selection of AU-based models of four conversational signals (“bored,” “confused,” “interested,” and “thinking”). Signaling and inferring these mental states is paramount to effective communication (*14*), especially in conversational settings (*15*). We identified five studies (*16*–*20*) that described their facial movements, coded them as AU combinations, and converted them into predictive models using our hypothesis kernel analysis method. Using an additional dataset of 2400 categorizations of the conversational signals from 40 participants (20 WE and 20 EA), we used the prediction-explanation-exploration framework to evaluate and optimize the conversational signal models just as we did with the basic emotion models. We found that most of the models accurately predict human categorizations but with a similar bias toward WE representations. As with the basic emotions, optimized and culture-aware models significantly improved prediction performance (still below noise ceiling), with less WE bias. In sum, these results replicate our findings for the basic emotions and demonstrate that our approach generalizes to other mental states.

## RESULTS

### Prediction

In this first stage, we used a new method to convert previously reported qualitative AU-based models of emotions into predictive models (see the “Hypothesis kernel analysis” section). We evaluated how well each model predicts the emotion category provided by humans performing the same task of categorizing a large set of randomly generated dynamic facial expressions (see the “Datasets used to evaluate models” section). We summarized how well each of the seven models predicts the categorization behavior of each of the 80 participants (40 WE and 40 EA) using the area under the receiver-operating curve (AUROC)—a metric with a chance level of 0.5 for a binary classification model (predicting one emotion versus all others; see Materials and Methods) that randomly assigns the labels and with a theoretical maximum score of 1 for a model that predicts each label perfectly. For each emotion, we also estimated a noise ceiling that represents the maximum achievable model performance (see the “Noise ceiling estimation” section). Maximum theoretical performance (i.e., AUROC = 1) implies that different participants categorize the same AU combinations with the same emotion labels. If participants categorize the same AU combinations with different emotion labels, then this “experimental noise” is irreducible by any model based solely on AUs, which reduces the noise ceiling below 1 and thus the proportion of variance that the model can explain accordingly.

**Fig. 3.**
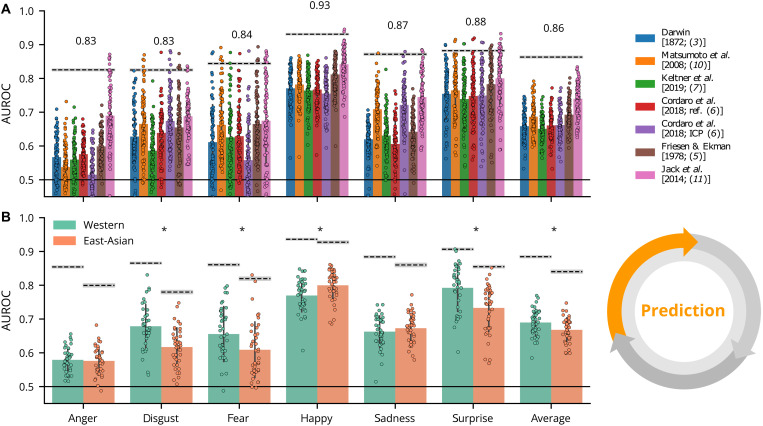
Prediction results. (**A**) Color-coded bars show the average AUROC score for each model (see legend at top) for each emotion separately. Dots represent the model predictions of each individual participant. Dashed lines represent the noise ceiling with specific values shown above (gray area represents ±1 SD based on bootstrapping repeated observations, see the “Noise ceiling estimation” section). The black solid line at the bottom represents chance performance (AUROC = 0.5). (**B**) The same AUROC scores are averaged across models and presented by culture. Asterisks indicate a significant AUROC score difference (*P* < 0.05; two-sided independent *t* test) across cultures.

Figure 3A summarizes the average prediction performance of each model as color-coded bars (see legend at top) for each emotion separately and with per-participant AUROC scores (color-coded dots). Dashed lines indicate the noise ceiling of each model (exact values shown above). Across most emotions, most models predict categorization behavior well above chance (i.e., an AUROC of 0.5) with some substantial differences between emotions—e.g., fear (average AUROC = 0.57) versus surprise (average AUROC = 0.76)—and between models—e.g., Keltner *et al.* [2019; (*7*); average AUROC = 0.66] versus Jack *et al.* [2014; (*11*); average AUROC = 0.74]. However, average performance (across models and emotions, AUROC = 0.68) is still well below the average noise ceiling (AUROC = 0.88), suggesting that the models do not perform optimally. Moreover, with a noise ceiling lower than the theoretical maximum (AUROC = 1), these AU-based models cannot, in principle, explain a nontrivial proportion of variation in human emotion categorizations.

Figure 3B shows performance differences (averaged across models) depending on participant culture (see fig. S1 for differences per model). On average, models performed significantly better (at α = 0.05) for WE participants than for EA participants for disgust (*t =* 4.07, *P* < 0.001, *d* = 1.07), fear (*t* = 2.48, *P* < 0.001, *d* = 0.56), and surprise (*t* = 3.81, *P* < 0.001, *d* = 0.87). In contrast, models performed better for EA participants than WE participants for happy (*t* = −2.87, *P* = 0.005, *d* = −0.65). It is important to note that these cross-cultural differences disappear at the exploration stage when the models comprise cultural accents.

### Explanation

In the second stage of the modeling cycle, we aim to explain the behavioral predictions and relative accuracy of the different models by quantifying the causal effect of each AU on model performance. To do so, we used the AU ablation method described earlier that systematically removes (i.e., “ablates”) individual AUs from each model and recomputes its behavioral prediction performance.

**Fig. 4.**
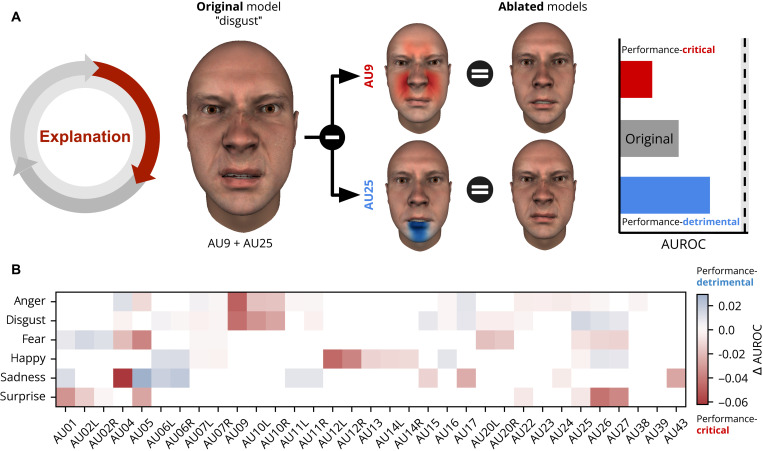
Explanation results. (**A**) Schematic visualization of the explanation process using an ablation method. The (hypothetical) original model shows the AUs associated with a given emotion—here, disgust is represented by AU9 and AU25. The ablated models show the remaining AUs following ablation of each individual AU. Performance-critical AUs (indicated in red) are those that decrease prediction performance when ablated, whereas performance-detrimental AUs (indicated in blue) are those that improve prediction performance relative to the original model (see color-coded bars to right; the vertical dashed line represents the noise ceiling). (**B**) Results of ablation analysis. The color-coded matrix shows the average difference in performance across all models when removing a given AU (*x* axis) for each emotion (*y* axis). Blue indicates that the AU ablation improves performance (performance-detrimental AUs); red indicates that it reduces performance (performance-critical AUs; see color bar to right). White cells show the AUs that were never part of any model (and thus could not be ablated).

Figure 4 shows how the method of ablating AUs from the facial expression models explains their predictions. Figure 4A schematizes the AU ablation procedure and the results it can yield. Specifically, for a particular model of disgust (AU9 + AU25, “original model”), ablation of an individual AU (e.g., AU9) may lead to a decrease or increase in model performance, thus indicating that the AU is performance-critical or performance-detrimental, respectively (see color-coded bar on right). We applied this ablation procedure to all models. Figure 4B shows the results as a color-coded matrix (see fig. S2 for the ablation results by culture). For each emotion category (*y* axis), the color-coded matrix shows the difference in AUROC performance according to the ablation of each individual AU (see *x* axis for labels), averaged across all models (fig. S3 shows results for individual models). Red indicates a decrease in the prediction of human behavior (e.g., AU9 for disgust and AU5 for surprise), and blue indicates increased performance (e.g., AU5 for sadness; see color bar at the right). Results show that each model of facial expression considered could potentially be improved by selectively adding performance-critical and removing performance-detrimental AUs (e.g., adding AU9 to the disgust models of Darwin [1872; (*3*)] and removing AU5 from the ‘sadness’ model of Cordaro *et al.* [2008; ref. (*6*)]. Furthermore, the ablation analyses in each culture show that their performance-critical and performance-detrimental AUs differ (see fig. S2), implying that exploration of culture-specific models could improve their prediction performance. To test this, we conducted the third and final exploration stage.

### Exploration

**Fig. 5.**
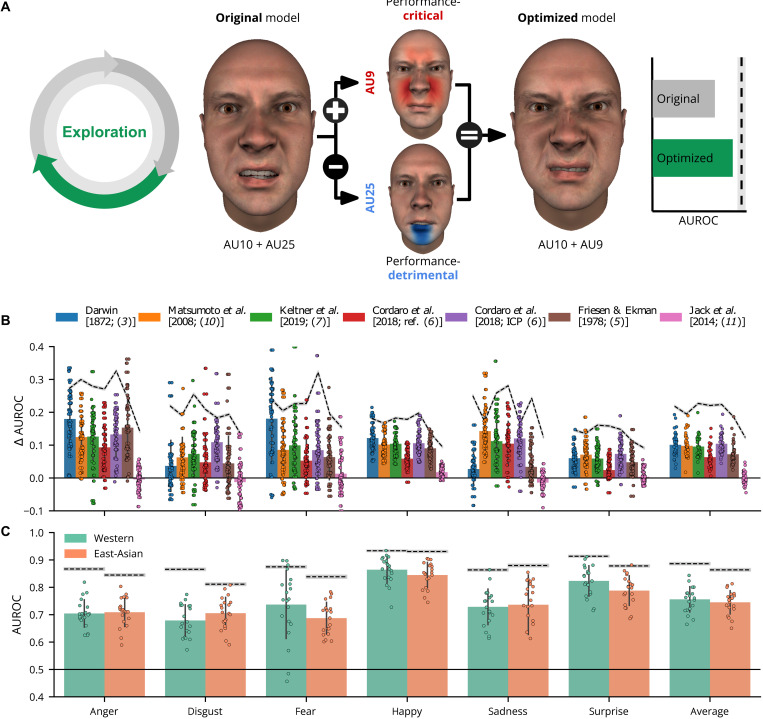
Exploration. (**A**) Schematic visualization of the exploration process, applied by enhancing the original models with additional performance-critical AUs (here, AU9, indicated in red) and removing performance-detrimental AUs (here, AU25, indicated in blue). (**B**) Each subplot shows the model performance increase (Δ AUROC) of the optimal model relative to the original model (cf. Fig. 3A). Dashed lines represent the original noise ceiling. (**C**) AUROC scores from the optimal culture-accented models, averaged across models and presented by culture. Performance did not significantly differ (at α = 0.05; two-sided independent *t* test) across cultures for any emotion (cf. Fig. 3B).

In this final stage of the modeling cycle, we aim to automatically generate and explore alternative, optimized models of facial expressions using the findings that explain human emotion categorizations. Because the set of performance-critical and performance-detrimental AUs are culture specific (see fig. S2), we explored model optimization separately in WE and EA cultures. Specifically, to optimize a given model in WE or EA culture, we (i) added all AUs that decreased performance when ablated (i.e., performance-critical AUs; represented in red in Fig. 4) and (ii) removed all AUs that increased performance when ablated (i.e., performance-detrimental AUs; represented in blue in Fig. 4). For each original expression model, this procedure yielded two optimized models: a WE-accented and an EA-accented model. Figure 5A illustrates this exploration procedure that results in an updated hypothetical model for disgust (AU10 + AU25) by adding a performance-critical AU (i.e., AU9) and removing a performance-detrimental AU (i.e., AU25). We then evaluated these optimized models on new (unseen) stimuli from new participants, effectively cross-validating the models (see the “Cross-validation” section for details).

Figure 5B shows, per emotion, the resulting improvement in predictive performance (represented as the ∆AUROC) of the optimized, culture-accented models relative to the original models (see color coding in legend at top; dashed line represents the irreducible noise ceiling). For most models and emotions, the automatic generation of optimized models improved performance, maximally for anger (median improvement = 0.12 across models) and minimally for surprise (median improvement = 0.05 across models). As shown in fig. S4, optimized models had better prediction performances because they better disentangled otherwise often confused emotions.

Last, Fig. 5C presents the predictions of the optimized, culture-accented models for WE and EA participants. As previewed when we presented the predictions of the original models in Fig. 3B, following exploration, the optimized, culture-accented models do not incur significant prediction differences between WE and EA participants (at α = 0.05). Therefore, these results demonstrate that exploring cultural accents with optimized models compensate for the WE bias of the models tested here (which replicate the results based on the original stimuli; see fig. S5). Additional analyses further demonstrate that culture-accented models result in less biased (fig. S6) and stronger prediction performance (see table S3) for most emotions relative to culture-agnostic models.

### Generalization to other emotions

**Fig. 6.**
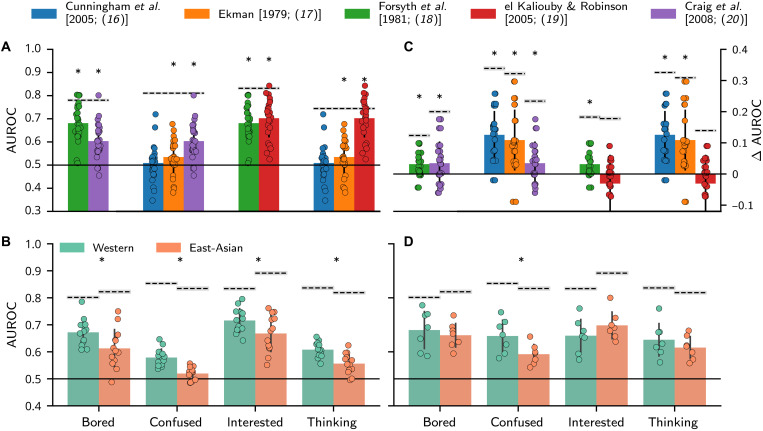
Original and optimized model performance for the conversational signal dataset. (**A**) Color-coded bars show the average AUROC score separately for each emotion. For each model, we only tested the categorizations that are covered by the model [e.g., only confused and thinking to evaluate Ekman, 1979 (*17*)]. In binary classifiers, the AUROC score is the same for each output class, which explains why AUROC scores are equal for the different emotions in the same model [this also applies to (C)]. (**B**) The AUROC scores of (A) are averaged across models and presented by culture. (**C**) Bars quantify the model performance increase (Δ AUROC) from the original (A) to the optimized models. (**D**) Average AUROC scores for the optimal models presented by culture. Black solid lines represent chance-level model performance; black dashed lines represent the noise ceiling. Asterisks indicate a significant AUROC score difference (*P* < 0.05; tested two-sided) against chance-level performance (A and C; one-sample *t* test) or between cultures (B and D; two-sample *t* test). See table S6 for detailed statistics of each *t* test.

In the prediction stage, we evaluated how well each model predicts human emotion categorizations. Figure 6A shows that all models explain a significant amount of variance of all emotions (see table S6 for detailed statistics of all tests shown in Fig. 6), except for Cunningham *et al.* [2005; (*16*)]. Moreover, Fig. 6B shows that the evaluated conversational signal models also perform significantly better with WE participants than EA participants, replicating the cultural bias observed with basic emotion models. In the explanation stage, an ablation analysis identified the performance-critical and performance-detrimental AUs for each model and emotion. The exploration stage used these insights to construct optimized, culture-accented models. Figure 6C outlines changes in predictive performance (Δ AUROC) of the optimized versus original models, showing that each model improved significantly, except el Kaliouby and Robinson [2005; (*19*)] which already performed close to the noise ceiling in the prediction stage (see Fig. 6A). As with the basic emotion models, the optimized conversational signal models do not perform significantly better or worse for either WE or EA participants—except for “confused” that still performed better for WE participants [*t*(10) = 2.42, *P* = 0.03, *d* = 1.40].

In sum, these results demonstrate how our framework can automatically generate and then explore updated, culture-accented models using insights gained from explaining prediction performance with performance-critical and performance-detrimental AUs. In turn, these optimized models can be empirically tested in additional human experiments.

## DISCUSSION

Since Darwin’s seminal work on the evolutionary origins of facial expressions of emotion (*3*), an important debate has centered on the question of which specific combinations of facial movements (i.e., AUs) best represent the six classic basic emotions. Here, we tested different models that offer competing hypotheses about how AUs specifically relate to facial expressions of emotions. After translating these qualitative models into predictive models with the novel technique of hypothesis kernel analysis, we embedded them into a new prediction-explanation-exploration framework. Within this framework, we compared how accurately each model predicts human emotion categorizations of a large set of dynamic facial expression stimuli from both WE and EA participants. We then explained which specific AUs causally affect prediction accuracy using a method of systematic AU ablation and lastly used these insights to automatically generate and explore updated models that capitalize on the relative strengths of the different models, to produce substantially improved predictive performance. Last, we showed that models of the six facial expressions with cultural accents better predicted the cultural diversity of human behavior. Our prediction-explanation-exploration cycle demonstrates that a model-based approach can summarize the strengths and limitations of each evaluated model and enables targeted and culturally aware improvements. Furthermore, we further validated the framework on additional facial expression categories (bored, confused, interested, and thinking) with similar results. We now discuss the implications of our study that can inform and guide knowledge developments in emotion research specifically and social cognition research more broadly.

### Toward formal models of facial expressions of emotions

In science, models are used to represent and reason about phenomena of interest. In the study of facial expressions of emotions, models aim to capture the way humans perceive and recognize emotions from faces. Over time, models have become increasingly refined, from Darwin’s verbal descriptions (*3*) to more systematic models of emotional facial expressions based on AUs that enhance their expressiveness (*5*–*7*, *10*). Such models have become competing hypotheses of the AUs that accurately represent emotional expressions. However, these models cannot be quantitatively tested or compared without a “common currency” to do so. As we showed, transforming them into predictive models offers such common currency in the form of predictive performance on independent data.

We showed that moving from qualitative and verbal to format and predictive models has three important advantages. First, predictive models allow us to precisely quantify what we can explain and we cannot (yet) explain, and using a model’s noise ceiling, we can gain insight into the model’s limitations. In the current study, for example, we showed that models that consider only AUs are unlikely to capture all variance in emotion categorizations, hinting at possible additional factors that influence facial expression perception (discussed below). Second, predictive models facilitate model comparison (*21*), which can generate important insights on why some models perform better than others. For example, Cordaro *et al.*’s [2018; international core pattern (*6*)] model best predicted disgust among the tested models but was worst for fear. Follow-up ablation analyses revealed that this model for fear lacked AU4 (Brow Lowerer), a performance-critical AU included in most other models. Adding this AU to the fear model of Cordaro *et al.* (*6*) drastically improved its predictive performance.

A third advantage is that predictive models enable rapid iteration between construction and evaluation of updated and more accurate models (*22*), as we did in the exploration stage. Such exercise can involve any modification to the model inputs or functional form. For example, updated models could refine their AU inputs by adding weights to each AU (to reflect their importance or probability) rather than using “on” versus “off” binary AUs as is standard or use the full AU time course as input instead of considering the peak AU amplitude only, as we did here. Given that AU onset (*11*, *23*) and speed (*24*) influence the emotional interpretation of facial expressions, additional temporal information about AUs is likely to improve model performance. Last, even more complex models that go beyond linear effects of AUs and consider nonlinear or interaction effects between AUs may prove beneficial (*25*, *26*).

One important implication using predictive models is that emotion research should move toward large (in terms of number of observations) and “rich” (in terms of the stimulus dimensions) datasets (*27*). Such datasets allow for better investigation of multiple competing high-powered and high-dimensional models. Our investigation illustrated this by testing and subsequently combining the strengths of multiple high-dimensional AU models on a stimulus set that broadly covers the domain of facial expressions. Note that the data and input to facial expression models, however, do not have to be restricted to facial movements. Below, we discuss how additional factors beyond facial movements can benefit facial expression models.

### Creating more granular facial expression models

We showed that even the best AU models cannot explain all the variance of emotion categorization behavior. Such suboptimal model performance indicates that models based on only AUs do not represent all the relevant information that humans process to infer emotions from faces. This is supported by studies showing that facial expression perception is influenced by factors beyond the expression itself, such as the (static) three-dimensional face of the expressor (*28*, *29*), the culture (*30*, *31*) and prior beliefs (*32*, *33*) of the perceiver, and the context of categorization (*34*, *35*), as highlighted in constructionist theories of emotion (*36*).

To gain a more fine-grained understanding of the information that is lacking, we computed a noise ceiling. Here, the noise ceiling shows that incorrect predictions of the model can be attributed either to missing/misspecified features of the expressive face or to perceiver-related individual differences. More granular facial expression models that incorporate these features could substantially improve the performance of facial expression models of emotions. One promising direction is to consider the face features that affect the static shape or complexion of the expressor, as we know that people incorporate such features into their facial expression judgements (*37*). For example, facial features statistically associated with particular cultures (such as skin color) have been shown to influence emotion perception (*38*), which may underlie the in-group advantage in emotion recognition [(*39*, *40*) but see (*41*)]. In addition, research showed that relatively masculine faces are more likely to be interpreted as angry and relatively feminine faces as happy (*42*, *43*). Beyond culture and sex, improved models could furthermore include other expressor-related features [such as age (*44*), perceived social traits (*45*), and social class (*46*)] that may affect categorization behavior.

Although additional expressor-related facial features may improve prediction performance, they cannot explain differences across individuals (i.e., the variance above the noise ceiling). Therefore, to explain this substantial amount of variance, we must turn to perceiver-related features, which could also be multiple, including the age, gender, sex, personality, and culture of the perceiver, all of which have been shown to influence the interpretation of facial expressions of emotion (*47*–*50*). Our study showed that the perceiver’s culture explains part of this variance and that our culture-aware models removed the initial bias towards WE cultures. Future models could incorporate more detailed cultural factors [such as Hofstede’s dimensions (*50*, *51*)] and other perceiver-related factors (*52*), which may reduce biases toward demographic groups overrepresented in emotion research (*27*, *53*).

### Generalization to a broader domain of affective, social, and communicative signals

We applied hypothesis kernel analysis and the prediction-explanation-exploration framework to both models of the six classic basic emotions, as well as models of conversational signals. Our applications were based on the availability of existing and competing AU-based models of facial expression of emotions (*13*, *53*). However, both hypothesis kernel analysis and the prediction-explanation-exploration framework can be used to model any affective, social, or communicative signal, as long as the inputs and model components are clearly and explicitly defined and operationalized (such as the FACS-based AUs in the current study).

One promising research direction is to apply the prediction-explanation-exploration framework to a larger set of emotions beyond the classic six emotions and four conversational signals we evaluated. Studies showed that people express and perceive many more emotions from faces (*13*, *54*), such as “doubt” and “awe,” including compound emotions [such as “happily surprised”; (*55*)]. Moreover, our framework also extends from categorical models to regression models of continuously varying signals—e.g., arousal, valence (*56*–*58*), dominance, and trustworthiness (*29*, *59*). Last, the range of applications is not limited to dynamic facial expression signals but can extend to static facial features [e.g., to model attractiveness (*60*)] and to dynamic and static body features (*61*), vocal features (*62*), and physiological features (*63*), given that the features are consistently and quantitatively defined [e.g., the “Body Action Coding System” (*64*)]. While quantitative models (*29*, *56*, *59*, *62*) exist for these different signals [reviewed in (*27*, *65*, *66*)], our framework can be used to further optimize these models and make them culturally sensitive.

To conclude, our hypothesis kernel analysis methodology and prediction-explanation-exploration framework enable the systematic testing and optimization of social signals. We found that individual models explain a substantial proportion of variance in emotion categorizations of both basic emotions and conversational signals. However, we demonstrated that combining the strengths of different models into updated, culturally aware models greatly improved model performance and reduced bias toward WE representations of emotions. The models’ noise ceiling revealed that models can likely be further improved by considering additional expressor-related and perceiver-related factors. We anticipate that our prediction-explanation-exploration framework in the context of predictive models will progress our understanding of social signaling, by developing models that more accurately reflect the complexity and diversity of human nonverbal communication.

## MATERIALS AND METHODS

### Hypothesis kernel analysis

To formalize the statistical relationship between AUs and emotion categories as predictive models, we propose a novel method we call hypothesis kernel analysis. We use this method to derive classification models that predict the probability of an emotion, given a set of AUs [analogous to how people infer emotions from facial expressions (*67*)]. What follows explains how the method works at a conceptual level. A detailed formal description is presented in the Supplementary Materials.

**Fig. 7.**
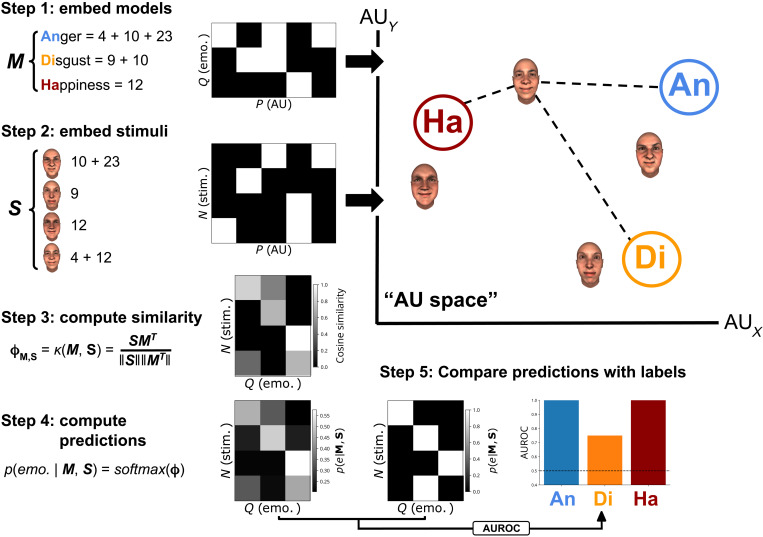
Schematic visualization of the proposed method. Here, we use a set of hypothetical AU-emotion relationships (***M***) and stimuli (***S***) based on a small set of AUs (five in total). The variable *P* represents the number of AUs, *Q* represents the number of emotions, and *N* represents the number of trials (here, facial expression stimuli). We illustrate the analysis in the two-dimensional space of two AUs, but in effect, the space is three-dimensional (33 AUs).

The idea of the hypothesis kernel analysis is to predict a categorical dependent variable (here, the perceived emotion) based on the similarity between an observation and a set of features (e.g., here, AUs, the independent variables) and a hypothesis (e.g., “happy is expressed with AUs 6 and 12”). We can compare this prediction to real observations to evaluate the accuracy of the hypothesis. Three methodological challenges must be overcome: (i) How should we measure the similarity between the observation and hypothesis? (ii) How should we derive a prediction based on this similarity? And (iii) how should we compare the predictions to real data? Figure 7 outlines a solution to the three challenges in five steps. We describe each one in turn.

1) Embed the hypothesis in AU space. We embed each hypothesis in a multidimensional space spanned by different AUs treated as variables. In this space, we represent the hypothesis of an emotion configuration (e.g., “happy = AU12 + 6”; ***M*** in Fig. 7) as a separate point (see step 1 in Fig. 7), which has as many coordinates as AUs. The value of the coordinates reflects the importance (or probability) of the AUs for the corresponding emotion. Note that each coordinate (i.e., AU) could take any value, but we use binary values (0: AU is not part of emotion, 1: AU is part of emotion) because the hypothesized models are binary.

2) Embed each stimulus in the same AU space as the hypotheses. To empirically test the hypothesized relationship between the AUs of a model and the emotion category, we embed the dynamic facial expression stimuli (e.g., AU12 + AU25 + AU17; ***S*** in Fig. 7) in the same multidimensional AU space. Each stimulus represents a single point (observation) in AU space, where its coordinates represent the AU amplitude (ranging from 0, not active, to 1, maximally active).

3) Compute the similarity between each stimulus and each hypothesized emotion category. With a kernel function (here, vector cosine, step 3 in Fig. 7), we quantify the similarity between the pairs of vectors (i.e., the stimuli, ***S***, and the models of the six emotions, ***M***; see table S3 for a comparison of model performance across different similarity and distance metrics).

4) Derive a prediction for each stimulus. To produce a probabilistic prediction of the emotion categories given a particular stimulus and hypothesis, we normalize the similarity values to the 0 to 1 range using the *softmax* function (step 4 in Fig. 7).

5) Quantify each model’s predictive performance. Each model’s predictive performance depends on the correspondence between its predictions and the actual participants’ emotion labels (see step 5 in Fig. 7). To quantify this correspondence, we used the AUROC as our model performance metric, because it is insensitive to class imbalance, allows for class-specific scores, and can handle probabilistic predictions. We report class-specific scores, which means that different emotions get separate scores with a chance level of 0.5 and a theoretical maximum of 1.

### Ablation and exploration analyses

To understand why some mappings perform better than others, we performed an ablation analysis, which removes (or ablates) AUs one by one from each model tested and then reruns the kernel analysis to recompute model performance. If an ablated AU decreases model performance for a given emotion on average across models, then this AU is critical for perceiving this emotion. We call such AUs “performance-critical.” Conversely, if an ablated AU increases performance for this emotion on average across models, then it is detrimental for perceiving this emotion and called a “performance-detrimental” AU.

Using the results from the ablation analyses, we explored “optimized” AU models. Specifically, for each model, we added all performance-critical AUs, removed all performance-detrimental AUs, and reran the predictive analysis for each optimized model separately. We then compared prediction performance of the original and optimized models. The optimized models were evaluated on a different subset of participants and trials than the participants and trials that were used for the ablation analysis (see the “Cross-validation” section).

### Noise ceiling estimation

Instead of interpreting model performance relative to a theoretical optimum, we used the noise ceiling, which estimates the explainable portion of variance in human behavior. Noise ceiling is used in systems neuroscience to correct model performance for noise in measured brain data and is typically applied in within-participant regression models (*8*). Here, we develop a method to compute noise ceilings for models with a categorical target variable (e.g., categorical emotion labels), applicable to within-participant and between-participant models [see also (*68*)]. We explain noise ceilings for classification models conceptually in this section. The Supplementary Materials provide a formal description.

Noise ceiling estimation adjusts the theoretical maximum performance of a predictive model to account for the presence of irreducible noise in the data. The noise ceiling imposes an upper bound on model performance (see Fig. 2). Here, we estimate a noise ceiling for the different AU models using the variance (or “inconsistency”) in emotion labels across participants in response to the same stimulus set. We use the noise ceiling to know whether the evaluated AU models are sufficiently accurate to explain variance that is explainable by AUs or whether we may need differently parameterized AU models. In addition, the “unexplainable” variance indicates how much of the variance in emotion labels is caused by factors other than AUs. This way, the importance and limitations of AUs can be empirically estimated.

### Evaluated models

The literature comprises many different AU-based models of facial expressions of basic emotions and, to a lesser extent, of conversational signals. We base our selection of basic emotion models on those summarized in table 1 of (*4*). In addition, we included the basic emotion model from the FACS manual [which we refer to as the “Friesen and Ekman, 1978” (*5*)] and an additional data-driven model [from (*11*); details follow below]. In addition, for our selection of conversational signal models, we identified five studies that contain hypotheses of facial movements associated with two or more signal categories (i.e., two from the following: bored, confused, interested, and thinking), which we translated to AUs.

The models propose that a number of AUs must be expressed to communicate a particular emotion. However, their comparison is complicated because not all of them posit a single set of AUs per emotion. Some contain multiple sets, such as Friesen and Ekman [1978; (*5*)] proposing that sadness can be expressed with AUs 1 + 4 or AUs 6 + 15. Others offer optional AUs for a set, such as Matsumoto *et al.* [2008; (*10*)] proposing that sadness is associated with AUs 1 + 15 and optionally with AUs 4 and/or 17. Last, some describe mutually exclusive options of AUs for a set, such as Matsumoto *et al.* [2008; (*10*)] proposing that “surprise” can be communicated with AUs 1 + 2 + 5 in combination with either AU25 or AU26.

To address this, we explicitly formulated all possible AU sets that communicate a particular emotion. For example, Matsumoto *et al.* [2008; (*10*)] propose that “disgust” is associated with AU9 or AU10 and, optionally, AU25 or AU26, which yields six different possible configurations (9, 10, 9 + 25, 9 + 26, 10 + 25, and 10 + 26). All AU configurations per basic emotion model are reported in Table 1; all AU configurations per conversational signal model are reported in table S5. Our analysis handles multiple sets per emotion, for each prediction separately, by using the set with the largest similarity to the stimulus under consideration (cf. steps 3 and 4 in Fig. 7). A simulation analysis demonstrates that this procedure does not unfairly advantage models with more sets per emotion (see fig. S7).

Furthermore, we added a basic emotion model based on the data-driven analysis from (*67*) [see also (*69*)]. We refer to this data-driven model as “Jack *et al.* (2014)” (*11*). For each AU and emotion, we computed the point-biserial Pearson correlation between the AU amplitudes and the binary emotion label (1 if this emotion was selected, 0 otherwise) for each participant. The raw correlations were averaged across participants and binarized on the basis of whether the correlation was statistically significant at α = 0.05 (1 if significant, 0 otherwise; uncorrected for multiple comparisons), which resulted in a binary 6 (emotion) × 33 (AU) mapping matrix. We chose this particular model estimation method (instead of, e.g., fitting a classifier directly to the data) because it yields a binary model matrix similar to those used in the theory-driven models, facilitating a fair comparison. We note that the data-driven models are estimated and evaluated on different partitions of the data, as explained in the next section.

### Cross-validation

**Fig. 8.**
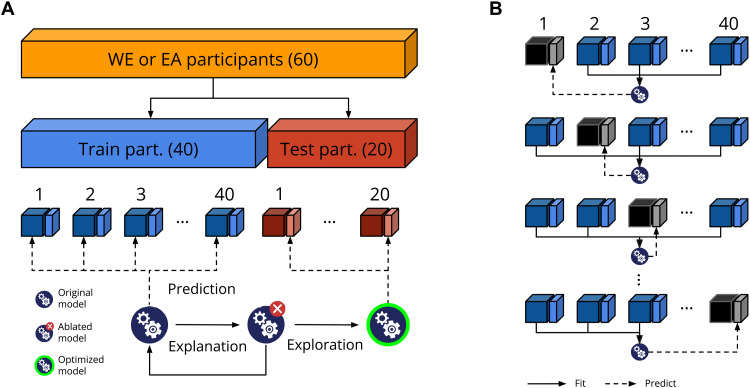
Cross-validation scheme used in the exploration stage. (**A**) For the theory-driven models, we divided the 60 participants per culture (WE and EA) into a train set (40 participants) and test set (20 participants). We further divided each participant’s trials into train trials (dark blue/dark red; 50%) and test trials (light blue/light red; 50%). We performed the prediction, explanation, and exploration stages on the train trials of the train participants (dark blue). At the exploration stage, we evaluate the optimized models on the test trials of the test participants (light red). (**B**) For the data-driven model in the prediction stage, we estimated and evaluated the data-driven models with a leave-one-participant-out cross-validation scheme within the train participants. We fitted emotion models using the train trials from *N*-1 participants and evaluated their predictive performance on the single left-out participant.

To avoid circularity in our analyses [also known as “double dipping” (*70*)], we cross-validated any step that involved optimization or fitting of models (see Fig. 8). Specifically, we performed the prediction and explanation stages on a subset of participants (40 per culture; 66.7%) and trials (50%), the “train set” (see Fig. 8A). In the exploration stage, we evaluated the optimized models on the left-out subset of participants (20 per culture; 33.3%) and trials (50%; the “test set”). That is, to avoid using the same data twice, we used different partitions of the data to construct the optimized models (using the train set) and to evaluate them (using the test set). The train and test sets contained data from new participants and new (unseen) stimuli. Specifically, the stimuli in the test set contained AU combinations and face identities that were not part of the train set. This effectively treats both participant and stimulus as random effects (*71*) and improves generalizability of the results (*72*).

The theory-driven models can be directly evaluated on the train set. In contrast, the data-driven models first must be estimated from the data, which requires cross-validation. To do so, we used a leave-one-participant-out cross-validation, iteratively estimating the data-driven models on *N*-1 participants (of the train set), using the estimated models to predict the test trials of the left-out participant (see Fig. 8B). Because leave-one-participant cross-validation yields a separate model for each participant, we aggregated all participant-specific models into the single aggregated model used in the explanation and exploration stages. For each aggregated emotion model, we included all AUs that were significant in at least one participant and weighted them by the proportion of participants for whom this AU was significant—e.g., in happy, if AU12 was significant in 90% of the participants, then its weight would be 0.9. We used this aggregated model in the same way as the “theory-driven” models (see Table 1) for the explanation and exploration stages.

### Datasets used to evaluate models

Our study uses two different datasets: one for categorizations of the six basic emotions and one for categorizations of what we refer to as “conversational signals.” We will refer to these datasets as the “basic emotion dataset” and the “conversational signal dataset,” respectively. The materials, experimental procedure, and data preprocessing procedure were similar for the two datasets, so the following descriptions apply to both datasets unless otherwise stated.

#### Participants

The basic emotion dataset contains data from 60 WE and 60 EA participants. The WE data have previously been used and described in (*11*, *73*) and included 59 European participants and 1 North American participant (31 female; mean age = 22 years, SD = 1.7 years). The EA data have previously been used and described in (*73*) and included 60 Chinese participants (24 female; mean age = 23 years, SD = 1.6 years). All WE participants were recruited in the United Kingdom and tested at the University of Glasgow; all EA participants were recruited in China and tested at the University of Electronic Science and Technology of China.

The conversational signal dataset contains data from 20 WE and 20 EA participants and has previously been used and described in (*15*, *74*). All WE participants were European (10 male; mean age = 21 years; SD = 2.3 years), and all EA participants were of Chinese nationality and heritage (10 male; mean age = 23 years; SD = 2.1 years). All participants were recruited in the United Kingdom and tested at the University of Glasgow. All participants lived in the United Kingdom, and all EA participants had U.K. residence of at most 3 months at the time of testing.

WE and EA participants (from both datasets) had all minimal experience with the other culture (as assessed by questionnaire; see the “Participant questionnaire” section in the Supplementary Materials), had normal or corrected-to-normal vision, and did not have any emotion-related atypicalities (autism spectrum disorder, depression, and anxiety), learning difficulties (e.g., dyslexia), synesthesia, or disorders of face perception (e.g., prosopagnosia) as per self-report. EA participants spoke proficient English (International English Language Testing System score ≥ 6.0, “competent user”). They gave written informed consent before testing and received £6 (WE and EA, basic emotion study) or ¥50 (EA, conversational signal study) per hour for their participation. The University of Glasgow College of Science and Engineering Ethics Committee provided ethical approval of the basic emotion study (reference ID 300160203) and the conversational signal study (reference ID 300140082).

#### Materials

Participants categorized 2400 short (1.25 s) video clips depicting a dynamic facial expression with a random combination of AUs. Each dynamic facial expression stimulus comprised one of eight “base faces” and was of the same ethnicity as the participant (WE base faces: four males, four females, mean age = 23 years, SD = 4.1 years; EA base faces: four males, four females, mean age = 22.1 years, SD = 1.0 years). Each face was animated with a subset of randomly selected AUs from a set of 42 possible AUs (with the number of AUs drawn from a binomial distribution with *n =* 5 and *P* = 0.6). The time course of each selected AU was determined by six parameters (onset latency, offset latency, peak latency, peak amplitude, acceleration, and deceleration), which were sampled from a uniform distribution from 0 to 1. All animations had a duration of 1.25 s (30 frames, presented at 24 frames/s). Our analyses only used the peak amplitude parameter and ignore the six other (temporal) parameters. The facial animations were rendered from frontal view using flat lighting, which avoids shadowing [see supplementary movie S1 from (*73*) for an example]. Although the AUs and their parameters were randomly sampled, the resulting facial expressions all displayed morphologically plausible facial movements, because the generative facial expression model prohibits impossible movements with morphological constraints [see (*69*) for details].

Participants viewed stimuli on a black background displayed on a monitor with a diagonal of 48.26 cm, a refresh rate of 60 Hz, and resolution of 1024 × 1280. Stimuli appeared in the central visual field, disappeared after the animation ended, and were followed by a black screen until the observer responded. To present each stimulus using the average visual angle of a human face (*75*) during typical social interaction (*76*), we used a chin rest to ensure a constant viewing distance of 68 cm (basic emotion study) or 71 cm (conversational signal study), with images subtending 14.25° (basic emotion study) or 15.24° (conversational signal study) visual angle vertically and 10.08° (basic emotion study) or 9.66° (conversational signal study) visual angle horizontally.

#### Procedure

In each experimental session, participants completed a seven-alternative forced-choice (basic emotion dataset) or five-alternative forced-choice (conversational signal dataset) emotion categorization task of 200 dynamic facial expression stimuli. Participants were instructed to label the stimuli with one of the six basic emotions (anger, disgust, fear, happy, sadness, and surprise; basic emotion study) or one of the four conversational signals (bored, confused, interested, and thinking; conversational signal study)—but only if the facial expression matched one of the emotion categories. Otherwise, they were instructed to respond “other.” Participants responded by clicking the response option using a mouse. After the emotion categorization (except when choosing other), participants were instructed to rate emotion intensity on a five-point scale from “very weak” to “very strong”; the intensity data are not used in the current study. Emotion labels were presented in the participant’s native language, i.e., either English (WE) or simplified Chinese (EA). The Chinese labels were provided by a professional translator using the double translation method (*77*), who confirmed that each matched the meaning of the corresponding English label.

Each participant completed the study in 12 different sessions across 3 to 5 days, with no more than three sessions per day and at least a 1-hour break between sessions. Each session lasted approximately 1 hour, including instruction and breaks. There was no evidence for drift or other changes in categorization behavior across the full duration of the experiment (see fig. S8).

#### Preprocessing

The original set of 42 AUs comprised 3 compound AUs (AU12 + 25, AU1 + 2, and AU6 + 12), 15 unilateral AUs (left or right, e.g., AU12L and AU12R), and 24 bilateral AUs (such as AU12). To encode each AU as an independent variable, we recoded compound AUs (e.g., AU1 + 2 as activation of both AU1 and AU2) and bilateral AUs (e.g., AU12 as activation of both AU12L and AU12R), yielding a total of 33 AUs: 1, 2L, 2R, 4, 5, 6L, 6R, 7L, 7R, 9, 10L, 10R, 11L, 11R, 12L, 12R, 13, 14L, 14R, 15, 16, 17, 20L, 20R, 22, 23, 24, 25, 26, 27, 38, 39, and 43 (where L = left and R = right).

We excluded the trials categorized as other from our analyses because there are no specific hypotheses about this category. For the basic emotion dataset, this leaves a grand total of 247,782 trials (total WE: 119,382, total EA: 128,400) with an average of 2065 trials per participant (average WE: 1990, average EA: 2140). This grand total contains 6473 repeated trials (total WE: 4658, total EA: 2322), i.e., stimuli with the same AUs and amplitudes, with an average of 38 repetitions per participant (average WE: 26, average EA: 55). For the conversational signal dataset, this leaves a grand total of 83,540 trials (total WE: 40,540, total EA: 43,000) with an average of 2089 trials per participant (average WE: 2027, average EA: 2150). This grand total contains 4134 repeated trials (total WE: 2314, total EA: 2322) with an average of 20 repetitions per participant (average WE: 18, average EA: 19).

## Notes

View/request a protocol for this paper from *Bio-protocol*.
