# Supplementary material for: Testing, explaining, and exploring models of facial expressions of emotions
Source: Sci Adv. 2023 Feb 10;9(6):eabq8421. doi: 10.1126/sciadv.abq8421 (PMC9916981; doi:10.1126/sciadv.abq8421)
Supplement: Supplementary file 1 — Supplementary Text Figs. S1 to S8 Tables S1 to S6 [file sciadv.abq8421_sm.pdf]

Science Advances
AAAS

Supplementary Materials for

Testing, explaining, and exploring models of facial expressions of emotions

Lukas Snoek *et al.*

Corresponding author: Lukas Snoek, lukassnoek@gmail.com

Sci. Adv. **9**, eabq8421 (2023)
DOI: 10.1126/sciadv.abq8421

This PDF file includes:

Supplementary Text
Figs. S1 to S8
Tables S1 to S6

Supplementary Text

Participant questionnaire

Each potential observer completed the following (culture appropriate) questionnaire. We selected only those answering "no" to all questions for participation in the experiment.
Western participants. Have you ever:

1. lived in a non-Western[1] country before (e.g., on a gap year, summer work, move due to parental employment)?
2. visited a non-Western country (e.g., vacation)?
3. dated a non-Westerner?
4. had a very close friendship with a non-Westerner?
5. been involved with any non-Western culture societies/groups?

East-Asian participants. Have you ever:

1. lived in a non-East Asian[2] country before (e.g., on a gap year, summer work, move due to parental employment)?
2. visited a non-East Asian country (e.g., vacation)?
3. dated a non-East Asian person?
4. had a very close friendship with a non-East Asian person?
5. been involved with any non-Eastern culture societies/groups?

[1] By Western groups/countries we are referring to Europe, North America, and United Kingdom.
[2] By East Asian groups/countries, we are referring to China, Japan, Korea, Mongolia, Thailand, and Taiwan.

Hypothesis kernel analysis

Below, we detail the methodology of hypothesis kernel analysis and noise ceiling estimation.

Step 1: Embedding hypotheses. First, we embed the model's hypotheses into a common space of AUs, where each AU is a dimension and each hypothesis (e.g., "disgust = AU9 + AU10") a single point in the space. For example, if we only considered a space of five AUs (AU4, AU9, AU10, AU12, and AU23), the hypothetical model of "disgust = AU9 + AU10", $\boldsymbol{M}_{\text{disgust}}$, would a point with five coordinates (i.e., a vector), whose values indicates the presence of a given AU as part (1) of the hypothesized configuration or not (0), as

$$\boldsymbol{M}_{disgust} = [0 \quad 1 \quad 1 \quad 0 \quad 0 \quad 0]$$

Note that AU values can also reflect their intensity, or probability. For example, if we hypothesize that disgust is a combination of AU9 at 100% intensity (or probability) but AU10 at 50% intensity (or probability), its embedding becomes:

$$M_{disgust} = [0 \quad 1 \quad .5 \quad 0 \quad 0 \quad 0]$$

Importantly, many studies outline models with hypotheses about *multiple* emotions (i.e. "classes"). Assume that our hypothetical model $\boldsymbol{M}$ comprises the six basic emotions, with hypothetical AU configurations as follows:

- anger = AU4 + AU5 + AU7
- disgust = AU9 + AU15
- fear = AU1 + AU2 + AU4 + AU7 + AU26
- sadness = AU1 + AU4 + AU15
- surprise = AU1 + AU2 + AU5 + AU26

This model $\boldsymbol{M}$, with C classes and D dimensions becomes the following $C \times D$ matrix,

$$\boldsymbol{M} = \begin{bmatrix} 0 & 0 & 1 & 1 & 0 & 1 & 0 & 0 & 0 & 0 \\ 0 & 0 & 0 & 0 & 0 & 0 & 1 & 0 & 1 & 0 \\ 1 & 1 & 1 & 0 & 0 & 1 & 0 & 0 & 0 & 1 \\ 0 & 0 & 0 & 0 & 1 & 0 & 0 & 1 & 0 & 0 \\ 1 & 0 & 1 & 0 & 0 & 0 & 0 & 0 & 1 & 0 \\ 1 & 1 & 0 & 1 & 0 & 0 & 0 & 0 & 0 & 1 \end{bmatrix}$$

where rows represent the C classes (here: six basic emotions) and the D columns represent the involved AUs.

Step 2: Embedding stimuli. In the previous section we outlined how to formalize AU-emotion models as points (or, equivalently, vectors) in AU space. One way to *evaluate* these formalized models is to evaluate them with actual categorical emotion labels from human participants in response to stimuli with known AU configurations.

In reference to our previously defined hypothetical 10-dimensional AU space, assume that we have categorical emotion labels e from a set of emotions E ($e \in E$) in response to a collection of N facial expression stimuli parameterized with random AU configurations, drawn from the same 10-dimensional AU space discussed before. With such data, we can encode the stimuli in AU space by quantifying each stimulus, $\boldsymbol{S}_i$, as a 10-dimensional "stimulus vector" containing nonzero values at positions associated with active AUs for that stimulus and zeros elsewhere. Note that, as is the case with model vectors, the stimulus vector's nonzero values at positions associated with active AUs can be all ones (if assumed to be equally "active") or be values proportional to the amplitude (or "activity") of the active AUs. For example, suppose that stimulus $\boldsymbol{S}_i$ contains AU1, AU5, and AU26 with amplitudes 0.1, 0.5, and 0.8 respectively

(assuming a maximum amplitude of 1). Then, formally, we can represent this stimulus, $\boldsymbol{S}_i$, as the following stimulus vector:

$$\boldsymbol{S}_i = [.1 \quad 0 \quad 0 \quad .5 \quad 0 \quad 0 \quad 0 \quad 0 \quad 0 \quad .8]$$

In case of multiple stimuli ($\boldsymbol{S}_1$, $\boldsymbol{S}_2$, ... $\boldsymbol{S}_N$), their model vectors can be vertically stacked in a single $N \times D$ "stimulus matrix", $\boldsymbol{S}$. Given that both a model ($\boldsymbol{M}$) and set of stimuli ($\boldsymbol{S}$) are encoded as matrices in the same D-dimensional AU space, we can discuss using kernels to generate quantitative predictors for stimuli given a particular theory.

Step 3: kernel functions. Kernel functions (κ) measure the similarity between two vectors. We use them to quantify the similarity ($\boldsymbol{\phi}$) between an AU stimulus ($\boldsymbol{S}_i$) and the AU model ($\boldsymbol{M}_j$) for a specific emotion (e.g., happy):

$$\phi_{ij} = \kappa(\boldsymbol{S}_i, \boldsymbol{M}_j)$$

Most linear kernel functions use a variant of the inner product between the two vectors. Here, we used the cosine kernel that normalizes the inner product between two vectors by the product of their norm:

$$\kappa(\boldsymbol{S}_i, \boldsymbol{M}_j) = \frac{\boldsymbol{S}_i \boldsymbol{M}_j^T}{\|\boldsymbol{S}_i\| \|\boldsymbol{M}_j\|}$$

Note that this model configuration is equivalent to a K-nearest neighbor classifier in which each AU vector ($\boldsymbol{M}_j$) functions as a neighbor (i.e., $K = C$). Also, Instead of using measures of similarity between two vectors (i.e., "kernels"), one could use measures of *distances* (δ_{ij}) between two vectors instead and subsequently invert it to get a similarity score again, i.e., $\phi_{ij} = \delta_{ij}^{-1}$. In practice, we find that it does not make much of a difference in terms of predictive performance (see Table S4).

Step 4: computing predictions. To generate a quantitative prediction for stimulus $\boldsymbol{S}_i$ (i.e., $\hat{\boldsymbol{e}}_i$), we still need a decision function that maps the data to a prediction. To generate a discrete prediction, we can take as prediction the emotion (across C classes) that maximizes its similarity of kernel function (κ), to the stimulus:

$$\hat{e}_i = \underset{j}{\operatorname{argmax}} \ \kappa(\boldsymbol{S}_i, \boldsymbol{M}_j)$$

In our analyses, we instead generate probabilistic predictions by normalizing the similarity vector with the *softmax* function. The resulting vector sums to 1 and its elements can be interpreted as probabilities (i.e., of an emotion given a stimulus and model matrix):

$$P(\boldsymbol{E}_j \mid \boldsymbol{M}, \boldsymbol{S}_i) = \frac{e^{\beta \phi_{ij}}}{\sum_{j=1}^{C} e^{\beta \phi_{ij}}}$$

where β is the "inverse temperature" parameter — a scaling parameter — which distributes relatively more mass onto the largest values within the sequence of similarities. We treat this parameter as a model hyperparameter (i.e., that could be manually tuned using cross-validation) that we set to 1.

Step 5: quantifying model performance. To evaluate the performance of each model, we compared their (discrete or probabilistic) emotion predictions for a set of stimuli with the actual participant responses. We quantified model performance, or "score", with function (q) that takes as inputs a set of predicted labels ($\hat{\boldsymbol{e}}$) and a set of "true" labels ($\boldsymbol{e}$) and returns the model performance:

$$\text{score} = q(\boldsymbol{e}, \hat{\boldsymbol{e}})$$

Instead of a single, class-average model performance estimate, some metrics return class-specific model performance scores. Our analyses use the "area under the curve of the receiver operating characteristic" (AUROC), which summarizes the quality of probabilistic predictions in a range from 0 to 1 (0.5 is chance level; 1 perfect prediction). We prefer AUROC because it applies to discrete and probabilistic predictions, it is insensitive to class imbalance (i.e., unequal frequencies of target classes), and it allows for class-specific performance estimates (for $C > 2$).

Noise ceiling estimation

Suppose that, for a given dataset, we find that a particular model yields a (class-average) AUROC score of 0.8 — what should we conclude from this score? It is above chance level (a score of 0.5) but also below perfect performance (i.e., a score of 1). Here, we interpret performance relative to a noise ceiling that represents an upper bound that incorporates the within- and between-participant variance in class labels. A noise ceiling estimates an upper bound for predictive models that is adjusted for the consistency of the target variable.

Noise ceiling partitions unexplained variance into in principle *explainable* variance (i.e., the noise ceiling minus the model performance) and *unexplainable* variance (i.e., the theoretical maximum performance minus the noise ceiling; see Figure 2). The amount of explainable variance in turn quantifies how much there is to gain for model improvement: if it is large, one might consider different or more complex or differently parameterized models; if it is small (i.e., the model performance is at or near the noise ceiling), one can conclude that the model cannot be improved any further (which does not mean that it is the *correct* model, however). With AU-emotion models, the noise ceiling illustrates the portion of variance in emotion inferences that can be explained by AUs.

Noise ceilings are routinely estimated in systems and cognitive neuroscience (*8, 9*). However, existing methods are limited to regression models that assume a continuous target variable, (e.g. a modality of brain measure). Our study deals with classification models, as we are trying to

predict a categorical target variable (i.e., categorical emotion labels). We propose a novel approach to estimate a noise ceiling for predictive performance of classification models.

To estimate a noise ceiling, we need repeated trials to estimate the variance (or, inversely, the "consistency") of the target variable and to estimate an upper bound on predictive performance. A noise ceiling formalizes the idea that a model can only perform as well as the consistency of responses. Trials are considered "repeats" if their representation in the model is the same — here, stimuli with the same AU configuration. Moreover, repeated trials may occur within- or between-participants, to estimate within- or between-noise a ceiling when working with a within- or between-participant model. Here, we only considered between-participant variance (as our dataset only contains between-participant repeats).

To illustrate noise ceiling computation for a between-participant model, consider the following minimal example. Suppose three participants labeled the same two facial expression stimuli ($\boldsymbol{S}_1$ and $\boldsymbol{S}_2$) as outlined in Table S7. The noise (*nc*) ceiling represents an upper bound of predictive performance for a given set of observations, the performance that an *optimal model* would obtain:

$$nc = q(\boldsymbol{e}, \boldsymbol{e}_{\text{optimal}})$$

Here, the optimal model can be any type of model constrained to make the same predictions for repeated observations. To a model, repeated observations represent identical input that should logically be identically predicted.

When predictions are discrete (i.e., a single label per trial), the optimal model predicts the mode across repeated observations. In our example, the optimal model would predict $\boldsymbol{S}_1$ as "Anger" and $\boldsymbol{S}_2$ as "Disgust." The noise ceiling is subsequently computed as the performance (e.g. with AUROC or simple accuracy metrics) of the optimal model given the true labels. In the example, the optimal model correctly predicts 2 out of 3 labels per stimulus, a class-average AUROC noise ceiling of 0.6667.

Discrete predictions can result in multiple modes (e.g., a given stimulus might be labeled "anger" in 50% of participants and "disgust" in the other 50% of participants). One could randomly pick one of these modes as the optimal prediction, which could arbitrarily impact the class-specific noise ceiling for the classes represented by the other modes. Alternatively, we suggest using probabilistic instead of discrete predictions. With probabilistic predictions, the optimal model does not predict the mode, but a probability distribution across labels equal to the proportion of each. For stimulus, $\boldsymbol{S}_i$, with R repeats, the probability of each class, $p(E_j)$, is computed as the proportion of labels, e_i, equal to that class:

$$p(E_j \mid S_i) = \frac{1}{R} \sum_{k=1}^{R} \begin{cases} 1, & e_{ik} = E_j \\ 0, & e_{ik} \neq E_j \end{cases}$$

In our example, the optimal prediction for each repetition of $\boldsymbol{S}_1$ is [0.667, 0.333] for "Anger" and "Disgust" and the optimal prediction for each repetition of $\boldsymbol{S}_2$ is also [0.667, 0.333]. The class-average AUROC noise ceiling would coincidentally be the same as with discrete predictions, 0.6667.

Supplementary Figures

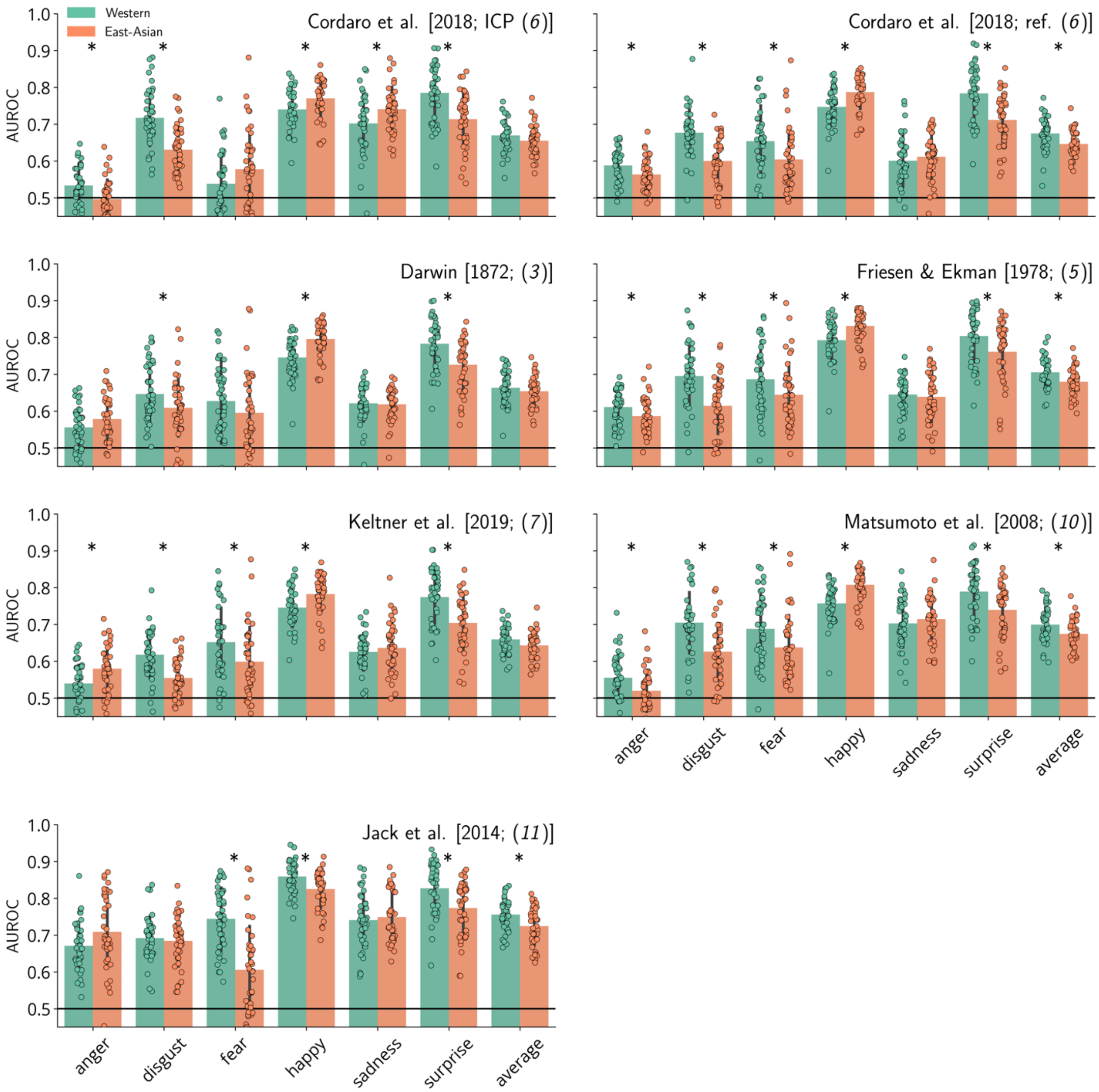

Fig. S1. Difference in performance between cultures, shown separately for each model. * indicates a statistically significant difference between cultures for a specific emotion (at $p < 0.05$; independent t-test).

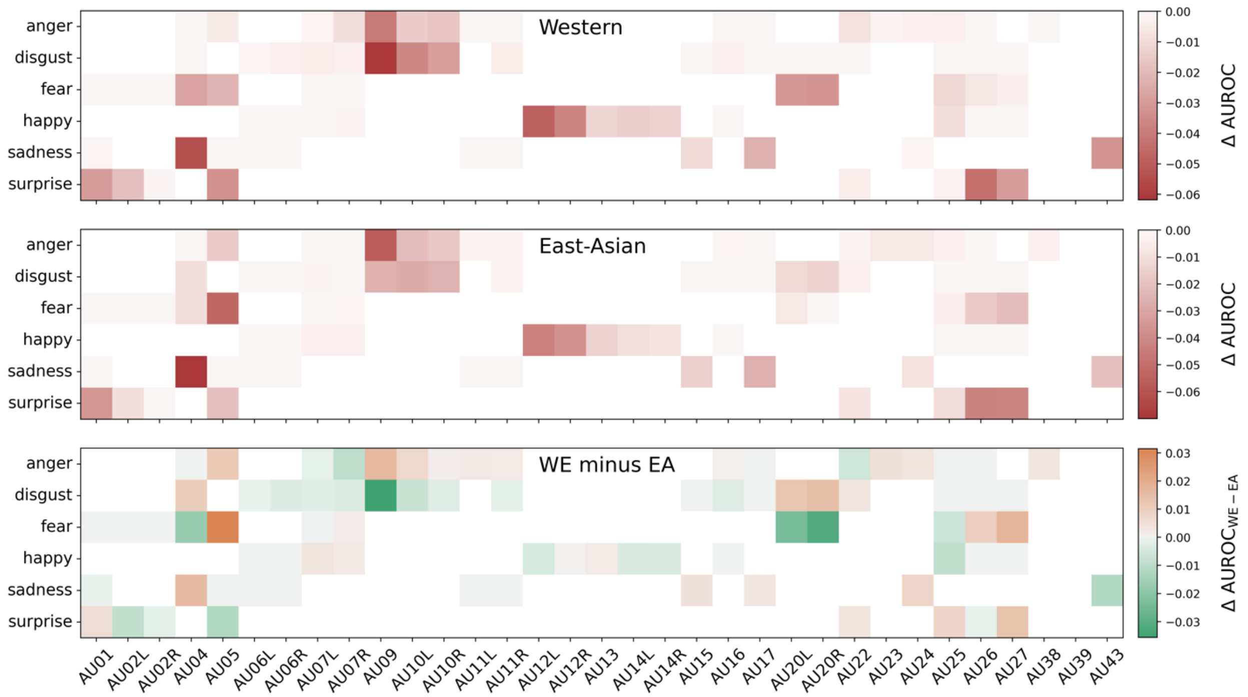

Fig. S2. Results from ablation analysis, visualized separately for Western (WE) participants (top), East-Asian (EA) participants (middle), and differences between WE and EA participants. For ease of interpretation, only the performance-critical AUs (i.e., AUs that decrease performance when ablated) are shown in the top and middle panel. In the panel with the differences between WE and EA, green cells indicate AUs that are more 'performance-critical' for WE participants than for EA participants and orange cells indicate AUs that are more performance-critical for EA participants than for WE participants.

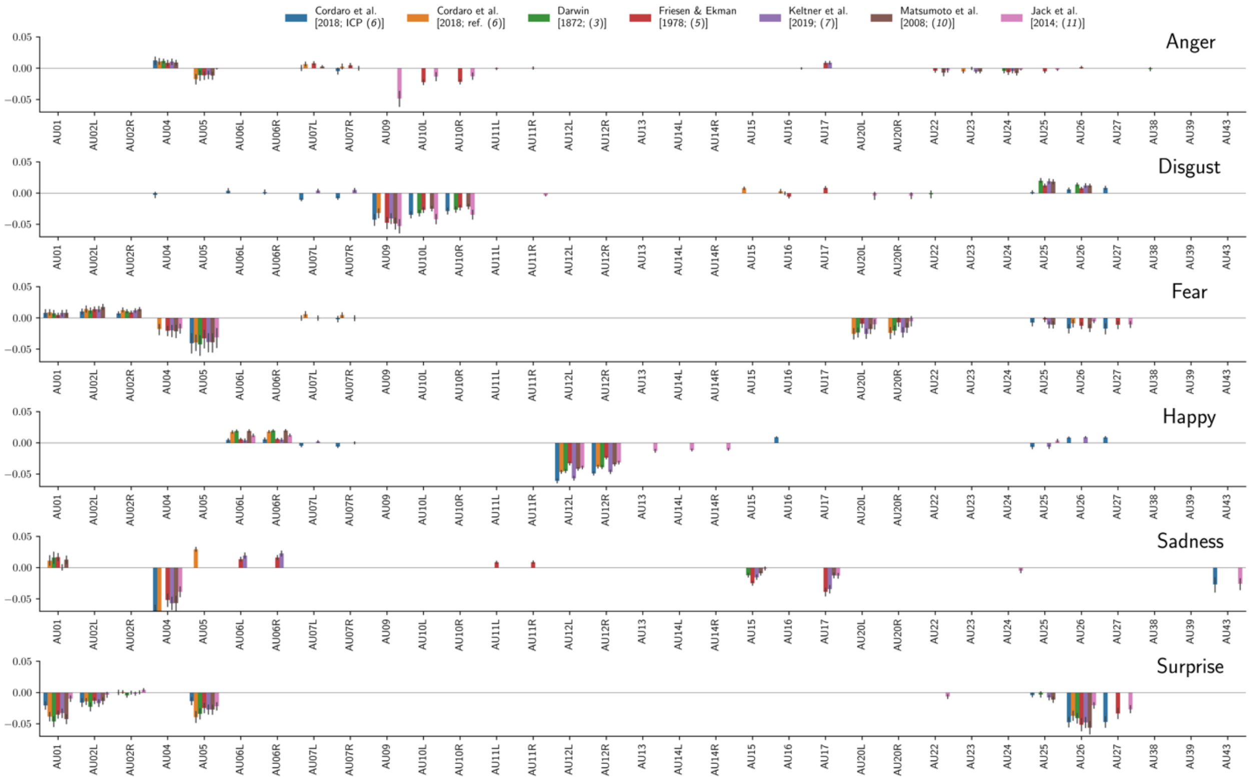

Fig. S3. Changes in model performance (AUROC) for each emotion and model after ablating an AU. Error bars indicate a 95% confidence interval obtained with 1000 bootstraps of the data.

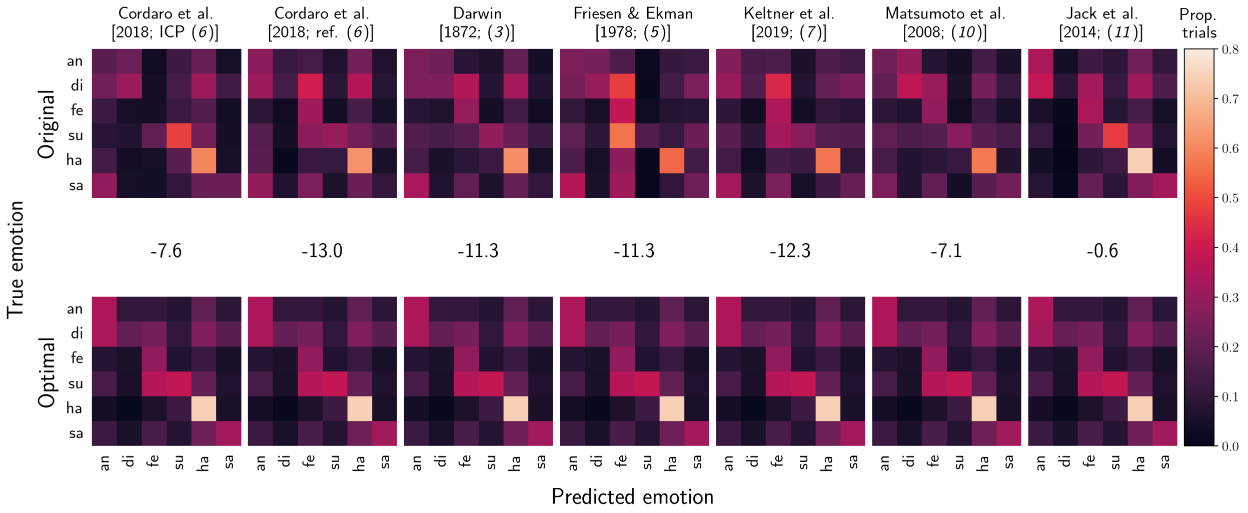

Fig. S4. Color-coded confusion matrices show the proportion of confusions with other emotions (off-diagonal cells) for the original (top row) and optimized models (bottom row) and accurate classification (diagonal cells). Brighter colors indicate a higher proportion of responses, darker colors indicate a lower proportion (see colorbar to right). For example, 'disgust' (second row) is often confused with 'fear' (third column), such as in Friesen & Ekman [1978; (*5*)] and Keltner et al. [2019; (*7*)]. The overall reduction in confusion rate (across emotions; in %) is presented between the confusion matrices of the original (top) and optimal (bottom) models. Prop. trials = proportion of trials.

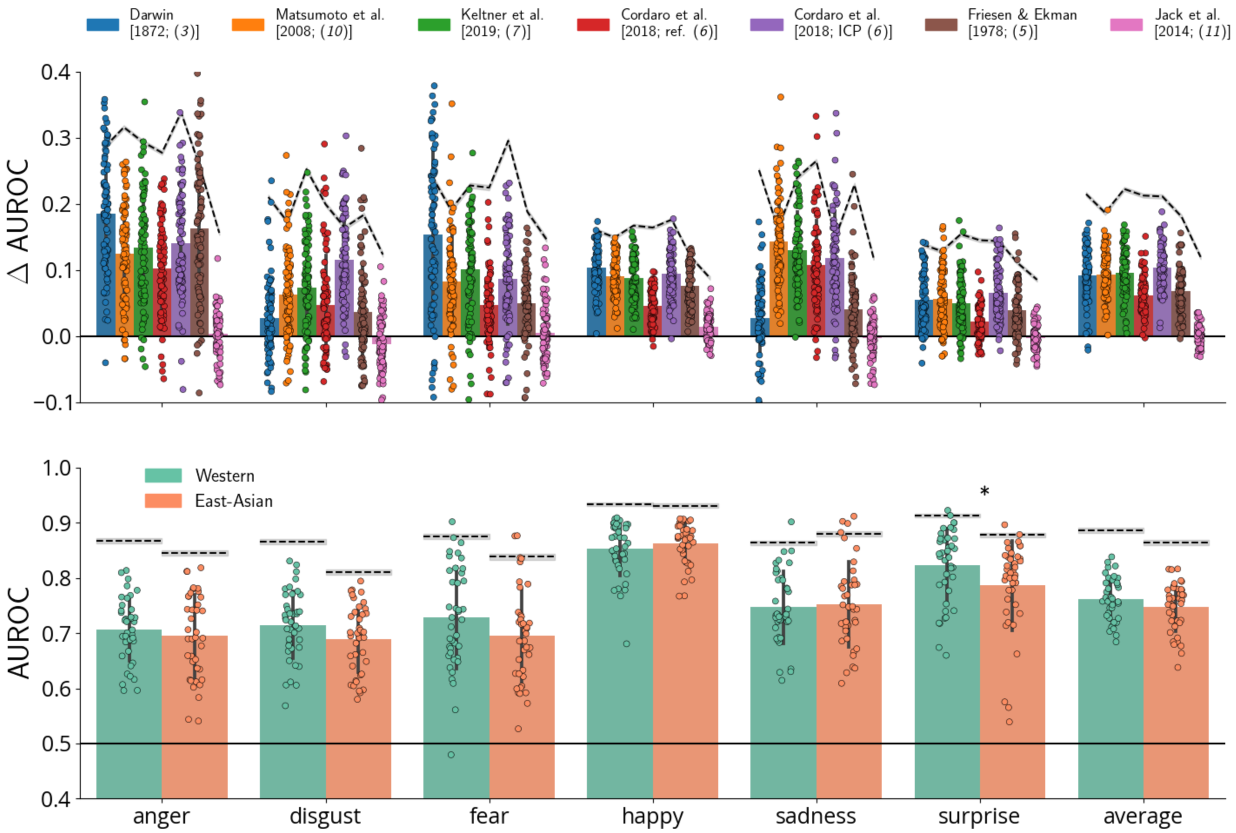

Fig. S5. Results of the optimized models on the *train* set (cf. Figure 5 in the main text showing results on the *test* set). Performance did not significantly differ (at α = 0.05; two-sided independent *t*-test) across cultures for any emotion except 'surprise,' $t(79) = 2.137$, $p = 0.036$, $d = 0.48$.

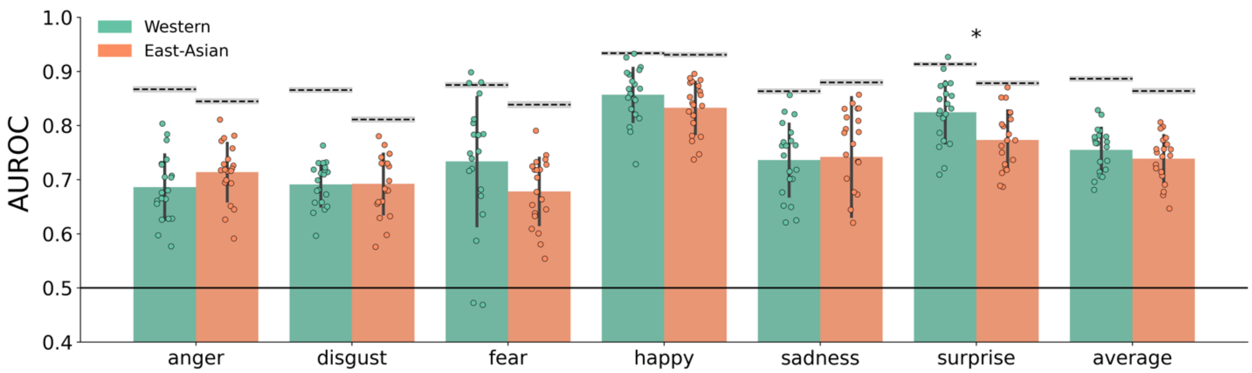

Fig. S6. Performance of optimized, *culture-agnostic* models, split by participant culture. Similar to the results obtained with optimized, culture-specific models (cf. Figure 5C), there are no significant differences in model performance between WE and EA participants for any emotion, with the notable exception of a significantly higher AUROC for WE participants than EA participants for surprise ($t = 2.86$, $p = 0.0069$, $d = 0.93$).

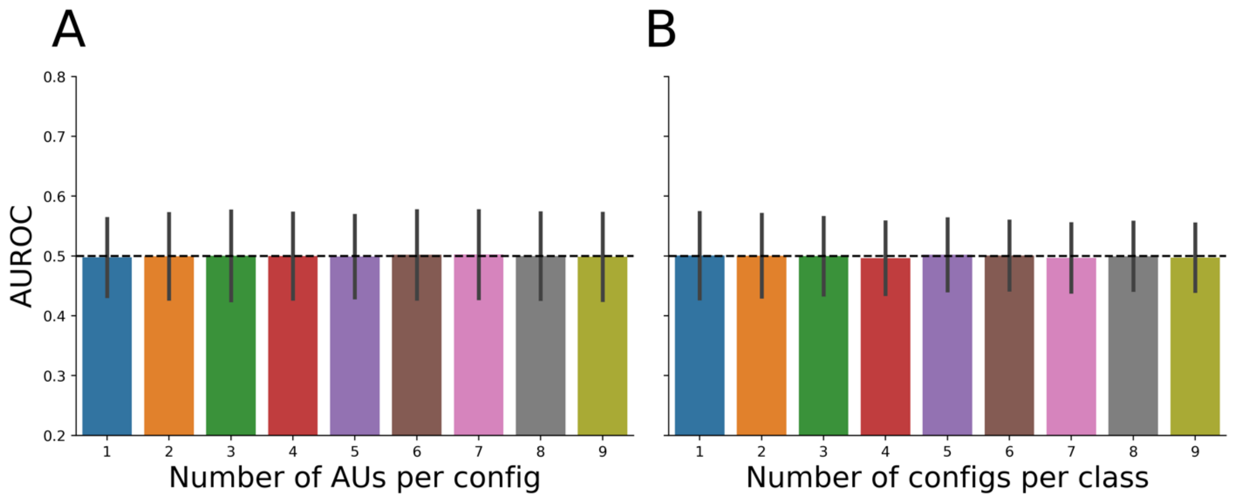

Fig. S7. Results from the simulation analysis with random model matrices in which the number of AUs per configuration (A) and the number of configurations per output class (B) were systematically varied. Bars represents the average AUROC score across 1000 simulations (error bars represent ±1 *SD*). None of the model matrices yielded contains more significant results than the expected amount given a significance threshold.

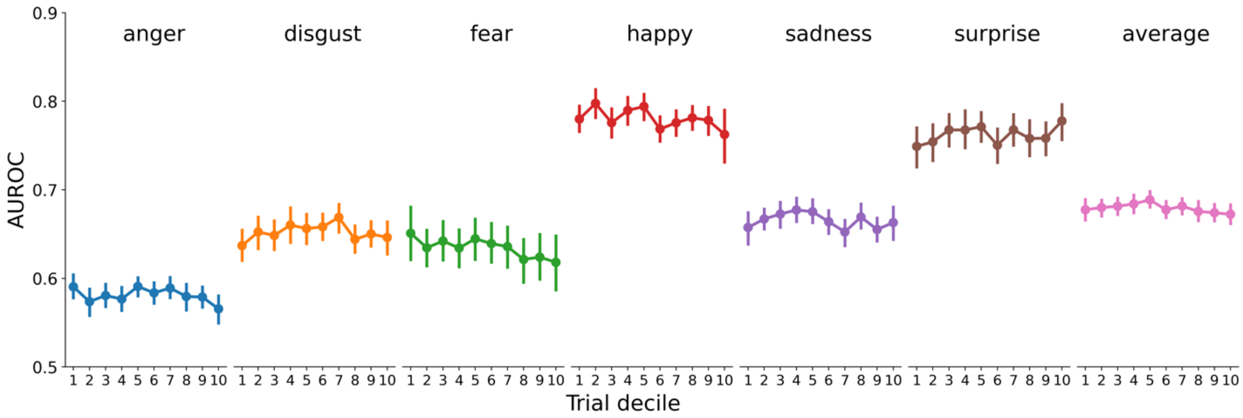

Fig. S8. Performance of the original (non-optimised) models, split by trial decile (x-axis; 1: first 10% of trials, 2: second 10% of trials, etc.). Comparing performance on the first half (decile 1-5) of the trials with the second half (decile 6-10) using a paired samples *t*-test (across 80 participants in the 'train set', $\alpha = 0.05$) shows no significant differences for any of the emotions.

Table S1. Common AUs to all optimized basic emotion models, shown separately for the culture-agnostic and culture-accented models (WE & EA).

Culture	Anger	Disgust	Fear	Happy	Sadness	Surprise
Agnostic	AU5, AU9, AU10L+R, AU11L, AU16, AU22, AU23, AU24, AU25, AU38	AU4, AU7L+R, AU9, AU10L+R, AU11R, AU16, AU20L+R, AU22	AU4, AU5, AU20L+R, AU25, AU26, AU27	AU7L+R, AU12L+R, AU13, AU14L+R, AU25	AU4, AU15, AU17, AU24, AU43	AU1, AU2L+R, AU5, AU22, AU25, AU26, AU27
WE	AU5, AU7L+R, AU9, AU10L+R, AU16, AU22, AU23, AU24, AU25	AU6L+R, AU7L+R, AU9, AU10L+R, AU11R, AU16, AU22	AU4, AU5, AU20L+R, AU25, AU26, AU27	AU7R, AU12L+R, AU13, AU14L+R, AU25	AU1, AU4, AU15, AU17, AU24, AU43	AU1, AU2L+R, AU5, AU22, AU25, AU26, AU27
EA	AU5, AU9, AU10L+R, AU11L+R, AU16, AU22, AU23, AU24, AU25, AU38	AU4, AU7L, AU9, AU10L+R, AU11R, AU20L+R, AU22	AU4, AU5, AU7R, AU20L+R, AU25, AU26, AU27	AU7L+R, AU12L+R, AU13, AU14L+R	AU4, AU15, AU17, AU24, AU43	AU1, AU2L, AU5, AU22, AU25, AU26, AU27

Table S2. Optimized performance (AUROC) of culture-agnostic models vs. culture-accented models in the exploration stage (relative to the original models). Statistics refers to the result of a two-sided paired samples *t*-test.

Emotion	Culture-ignorant		Culture-accented		*t*-value (*df* = 39)	*p*-value	Cohen's *d*
	M	*SD*	*M*	*SD*			
Anger	.108	.066	.115	.058	-1.77	.085	-0.11
Disgust	.051	.056	.052	.063	-0.05	.959	-0.01
Fear	.077	.068	.083	.069	-2.05	.047	-0.09
Happy	.076	.028	.086	.029	-8.35	<.000	-0.35
Sadness	.082	.051	.075	.048	4.03	<.000	0.14
Surprise	.042	.027	.049	.031	-3.37	.002	-0.23
Average	.073	.025	.076	.027	-2.38	.022	-0.15

Table S3. Performance of the evaluated models for different kernel functions.

Model/emotion	Cosine	Euclidean	L1	L2	Linear	Sigmoid
Darwin [1872; (*3*)]						
Anger	0.567	0.570	0.571	0.570	0.563	0.558
Disgust	0.628	0.630	0.628	0.630	0.620	0.619
Fear	0.611	0.614	0.616	0.614	0.609	0.606
Happy	0.771	0.769	0.769	0.769	0.767	0.768
Sadness	0.620	0.632	0.636	0.632	0.644	0.641
Surprise	0.754	0.737	0.726	0.737	0.751	0.748
Matsumoto et al. [2008; (*10*)]						
Anger	0.537	0.525	0.508	0.525	0.568	0.558
Disgust	0.666	0.621	0.609	0.621	0.652	0.657
Fear	0.662	0.650	0.630	0.650	0.656	0.659
Happy	0.782	0.778	0.777	0.778	0.771	0.773
Sadness	0.709	0.677	0.682	0.677	0.698	0.705
Surprise	0.765	0.736	0.710	0.736	0.755	0.754
Keltner et al. [2019; (*7*)]						
Anger	0.560	0.554	0.553	0.554	0.575	0.563
Disgust	0.586	0.573	0.571	0.573	0.592	0.582
Fear	0.626	0.614	0.600	0.614	0.629	0.629
Happy	0.764	0.757	0.760	0.757	0.757	0.753
Sadness	0.631	0.632	0.634	0.632	0.641	0.628
Surprise	0.739	0.734	0.735	0.734	0.729	0.736
Cordaro et al. [2018; ref. (*6*)]						
Anger	0.576	0.570	0.564	0.570	0.588	0.572
Disgust	0.639	0.616	0.611	0.616	0.617	0.625
Fear	0.629	0.607	0.588	0.607	0.639	0.639
Happy	0.767	0.767	0.767	0.767	0.757	0.761
Sadness	0.606	0.630	0.636	0.630	0.622	0.621
Surprise	0.748	0.742	0.739	0.742	0.735	0.742
Cordaro et al. [2018; ICP (*6*)]						
Anger	0.515	0.518	0.526	0.518	0.515	0.511
Disgust	0.674	0.636	0.598	0.636	0.678	0.651
Fear	0.558	0.565	0.562	0.565	0.560	0.558
Happy	0.755	0.733	0.726	0.733	0.753	0.747
Sadness	0.722	0.708	0.702	0.708	0.715	0.717
Surprise	0.749	0.739	0.729	0.739	0.731	0.741
Friesen & Ekman [1978; (*5*)]						
Anger	0.599	0.529	0.522	0.529	0.633	0.630
Disgust	0.655	0.650	0.645	0.650	0.631	0.637
Fear	0.665	0.614	0.613	0.614	0.651	0.659
Happy	0.812	0.816	0.816	0.816	0.790	0.791
Sadness	0.642	0.613	0.622	0.613	0.664	0.651
Surprise	0.783	0.730	0.730	0.730	0.760	0.764
Jack et al. [2014; (*11*)]						
Anger	0.696	0.680	0.681	0.680	0.684	0.690
Disgust	0.717	0.710	0.700	0.710	0.702	0.707
Fear	0.704	0.679	0.682	0.679	0.671	0.684
Happy	0.843	0.826	0.826	0.826	0.827	0.829
Sadness	0.753	0.747	0.745	0.747	0.750	0.755
Surprise	0.807	0.788	0.780	0.788	0.798	0.801

Note: The cosine, sigmoid, and linear kernels are measures of similarity, but the Euclidean, L1, and L2 kernels measure distance. For these distance functions, the distances were converted to similarities by inverting them. Only the train data was used to evaluate the different kernels.

Table S4. Evaluated conversational signal models in our study.

Emotion category	Cunningham et al. [2005; (*16*)]	Ekman [1979; (*17*)]	Forsyth et al. [1981; (*18*)]	el Kaliouby & Robinson [2005; (*19*)]	Craig et al. [2008; (*20*)]
Bored	4		None		43
Confused	4 + 20	- 4<br>- 1 + 2			- 4 + 7<br>- 4 + 7 + 12
Interested			1 + 2 + 6 + 12	1 + 2 + 12 + (26 ∨ 27)	
Thinking		1 + 2 + 4		23 + 24	

Note: The + symbol means that AUs occur together. The ∨ symbol represents "or", so e.g. (26 ∨ 27) means that either AU25 or AU26 may be included in the configuration. When multiple configurations are explicitly proposed for a given emotion, they are represented as separate bullet points (-). 'None' means that the emotion is represented by an absence of any AU.

Table S5. Statistics reported in Fig. 6 in the main text.

Panel	Model	Emotion	*t*-value (dof = 10)	*p*-value	Cohen's *d*
6A	Forsyth et al. [1981; (*18*)]	bored/interested	11.86	<0.001	2.33
	Craig et al. [2008; (*20*)]	bored/confused	6.92	<0.001	1.36
	Cunningham et al. [2005; (*16*)]	confused/thinking	0.61	0.550	0.12
	Ekman [1979; (*17*)]	confused/thinking	2.35	0.027	0.46
	el Kaliouby & Robinson [2005; (*19*)]	interested/thinking	12.43	<0.001	2.44
6B	Average	bored	2.44	0.022	1.00
	Average	confused	5.31	<0.001	2.17
	Average	interested	2.096	0.047	0.86
	Average	thinking	3.91	<0.001	1.60
6C	Forsyth et al. [1981; (*18*)]	bored/interested	4.28	<0.001	0.81
	Craig et al. [2008; (*20*)]	bored/confused	2.63	0.014	0.50
	Cunningham et al. [2005; (*16*)]	confused/thinking	8.70	<0.001	1.64
	Ekman [1979; (*17*)]	confused/thinking	5.90	<0.001	1.11
	el Kaliouby & Robinson [2005; (*19*)]	interested/thinking	-1.76	0.090	-0.33
6D	Average	bored	0.58	0.570	0.34
	Average	confused	2.42	0.032	1.40
	Average	interested	-1.21	0.250	-0.70
	Average	thinking	0.99	0.343	0.57

Note: 'panel' refers to the panel (A, B, C, D) of Figure 6 in the main text. For panel 6A and 6C, the statistics for the two listed emotions are the same because AUROC is the same for both classes in a binary (i.e., two-class) classification context. For the statistics in panel 6A and 6C, a one-sample *t*-test was used against a population mean of 0.5 (chance-level AUROC); for the statistics in panel 6B and 6D, a two-sample *t*-test was used to test the difference in performance for WE and EA participants.

Table S6. Hypothetical emotion labels from three participants in response to two stimuli.

Stimulus	Labels participant 1	Labels participant 2	Labels participant 3	Optimal pred.
S_1	Anger	Disgust	Anger	Anger
S_2	Disgust	Disgust	Anger	Disgust
